# Mitochondrial dysfunction and autism: comprehensive genetic analyses of children with autism and mtDNA deletion

**DOI:** 10.1186/s12993-018-0135-x

**Published:** 2018-02-20

**Authors:** Noémi Ágnes Varga, Klára Pentelényi, Péter Balicza, András Gézsi, Viktória Reményi, Vivien Hársfalvi, Renáta Bencsik, Anett Illés, Csilla Prekop, Mária Judit Molnár

**Affiliations:** 1 Institute of Genomic Medicine and Rare Disorders, Semmelweis University, Tömő Str. 25-29, Budapest, 1083 Hungary; 2 Department of Genetics, Cell- and Immunobiology, Semmelweis University, Nagyvárad tér 4, Budapest, 1089 Hungary; 3 Vadaskert Foundation for Children’s Mental Health, Lipótmezei Str. 1-5, Budapest, 1021 Hungary

**Keywords:** Autism, Mitochondrial dysfunction, mtDNA deletion, ASD associated genetic alterations, Intergenomic communication

## Abstract

**Background:**

The etiology of autism spectrum disorders (ASD) is very heterogeneous. Mitochondrial dysfunction has been described in ASD; however, primary mitochondrial disease has been genetically proven in a small subset of patients. The main goal of the present study was to investigate correlations between mitochondrial DNA (mtDNA) changes and alterations of genes associated with mtDNA maintenance or ASD.

**Methods:**

Sixty patients with ASD and sixty healthy individuals were screened for common mtDNA mutations. Next generation sequencing was performed on patients with major mtDNA deletions (mtdel-ASD) using two gene panels to investigate nuclear genes that are associated with ASD or are responsible for mtDNA maintenance. Cohorts of healthy controls, ASD patients without mtDNA alterations, and patients with mitochondrial disorders (non-ASD) harbouring mtDNA deletions served as comparison groups.

**Results:**

MtDNA deletions were confirmed in 16.6% (10/60) of patients with ASD (mtdel-ASD). In 90% of this mtdel-ASD children we found rare SNVs in ASD-associated genes (one of those was pathogenic). In the intergenomic panel of this cohort one likely pathogenic variant was present. In patients with mitochondrial disease in genes responsible for mtDNA maintenance pathogenic mutations and variants of uncertain significance (VUS) were detected more frequently than those found in patients from the mtdel-ASD or other comparison groups. In healthy controls and in patients without a mtDNA deletion, only VUS were detected in both panel.

**Conclusions:**

MtDNA alterations are more common in patients with ASD than in control individuals. MtDNA deletions are not isolated genetic alterations found in ASD; they coexist either with other ASD-associated genetic risk factors or with alterations in genes responsible for intergenomic communication. These findings indicate that mitochondrial dysfunction is not rare in ASD. The occurring mtDNA deletions in ASD may be mostly a consequence of the alterations of the causative culprit genes for autism or genes responsible for mtDNA maintenance, or because of the harmful effect of environmental factors.

**Electronic supplementary material:**

The online version of this article (10.1186/s12993-018-0135-x) contains supplementary material, which is available to authorized users.

## Background

In recent years, the number of patients diagnosed with autism spectrum disorders (ASD) has increased with current studies reporting a prevalence of 1% [1]. ASD shows extreme clinical heterogeneity; however, the diagnosis of ASD according to the Diagnostic and Statistical Manual of Mental Disorders (5th edition) is based on deficits in two areas—social communication and restricted, repetitive behaviour or interests. The patient must have deficits in both areas, and symptoms must be present from early childhood [2]. The genetic architecture of ASD is very diverse consisting of a variety of genetic alterations, such as chromosomal abnormalities, copy number variations, rare single nucleotide variants (SNVs), common polymorphic variations, and epigenetic modifications; however, only 6–15% of children with ASD have well-defined genetic syndromes [3]. Because of the development of high-throughput sequencing methods, many highly penetrant genetic causes of ASD have been identified, but the underlying genetic background of 70% of cases remains unexplained [4].

Mitochondrial disease (MD) is presently one of the most recognized metabolic diseases caused by the failure of both nuclear and/or mitochondrial DNA (mtDNA). The prevalence of mtDNA mutations responsible for MD is 1 in 5000, whereas that of nuclear mutations is 2.9 per 100,000 cases [5]. Although MD frequently results in a spectrum of disorders with multisystemic presentations, neurological symptoms are common because tissues with high-energy demands, such as neural tissue, are often the most strongly affected by mitochondrial dysfunction. Even though the diagnosis of MD is increasing and becoming more frequent, the exact genetic background in many cases remains unconfirmed. Mitochondrial dysfunction can be caused by either primary MD or secondary mitochondrial damage [6]. Primary MD is because of genetic defects in mtDNA or a defect in a nuclear gene that is important for mitochondrial function. These mutations usually affect proteins involved in reactions of oxidative phosphorylation (OXPHOS). However, many disorders show similar effects in terms of mitochondrial dysfunction, but are elicited by mutations in other genes not related directly to normal mitochondrial function [7]. In other cases environmental factors, associated disorders or ageing are resulting in secondary alterations.

Several authors have proposed that mitochondrial dysfunction may be one of the most common medical conditions associated with autism [8, 9]. Lombard et al. [10] proposed that ASD may be a condition with abnormal mitochondrial function. Clinical and biochemical studies have uncovered an emerging link between mitochondrial dysfunction and neurodevelopmental disorders, including intellectual disability [11], childhood epilepsy, and ASD [9]. Furthermore, mitochondrial dysfunction has been associated with some forms of syndromic ASD [8, 11]. In many of these studies, biochemical changes, such as elevated levels of creatine kinase, lactate, pyruvate, carnitine, ammonia, and alanine were detected in the serum of patients with ASD [11–14]. In other studies, altered respiratory chain enzyme activities [15] or decreased expression of OXPHOS genes were detected in autistic brain [16], findings which indicate abnormal or altered mitochondrial function.

Damage to the OXPHOS system was found in individuals with ASD by Napoli et al. [17] and reviewed by Valenti et al. [11]. Oliveira et al. [14] found that 7% (7/100) of children with ASD, who were clinically indistinguishable from other affected children with ASD, exhibited a mitochondrial respiratory chain disorder. Weissman et al. [18] proposed that defective mitochondrial OXPHOS may be an additional underlying pathogenic mechanism in a subset of individuals with autism.

Despite evidence of altered mitochondrial function in some individuals with ASD, it is not known whether mitochondrial dysfunction is a cause or an effect of ASD. Although a mitochondrial subgroup in ASD could be identified [19], findings from review articles, such as those of Palmieri and Persico [19] and Rossignol and Frye [9], found that even in this subgroup the causative genetic factor could be identified in a proportion of cases (23%). In cases of non-syndromic ASD, mitochondrial dysfunction without mtDNA alterations has been frequently observed [8, 9]. In a systematic review and meta-analysis, Rossignol reported that MD was present in 5% of children with ASD [9], and in this ASD/MD subgroup, mtDNA abnormalities were found in 23% of patients [9]. These findings demonstrate that primary MD may be present in a subgroup of children with ASD.

Some studies have reported mtDNA deletions in individuals with ASD [12, 20–22]. Single mtDNA deletions have a role in different paediatric and adult onset primary MDs such as Kearns–Sayre syndrome, Pearson syndrome, and progressive ophthalmoplegia externa [23]. Multiple mtDNA deletions occur mostly because of pathogenic mutations in genes responsible for intergenomic communication; however, they are often related to ageing or harmful environmental factors as well because mtDNA has a poor DNA repair system [6, 24].

The aim of the present study was to investigate the presence of the most common pathogenic mtDNA alterations in patients with ASD and to elucidate the etiology of these mtDNA alterations by analysing their co-occurrence with both known ASD-associated genes and genes responsible for mtDNA maintenance and by comparison the targeted NGS data (ASD associated genes and genes responsible for mtDNA maintenance) of cases with and without mtdel-ASD, patients with primary mitochondrial disorders and healthy controls.

## Methods

### Patients

Detailed clinical examinations consisting of a general medical examination and neurological assessment were performed. A diagnosis of ASD was made using the ADI-R (autism diagnostic interview—revised) and ADOS (autism diagnostic observation schedule). Patients were screened for minor physical abnormalities, which were selected based on the Méhes Scale [25]. Family history and detailed environmental/societal data were collected from the first degree relatives of each patient. Any disorders present in the parents as well as environmental factors were registered. Written informed consent was obtained from the parents of the patient. This study was performed in accordance with the Helsinki Declaration of 1975 and was approved by the Hungarian Research Ethics Committee (44599-2/2013/EKU). The diagnosis of ASD was based on the standardized ADI-R in Hungarian, which was published by the Autism Foundation (Kapocs Publisher), according to the following scores: A ≥ 10 (social interaction), B ≥ 7 (communication), C ≥ 3 (repetitive stereotype manner), D ≥ 1 (abnormal development under 36 months). Sixty children with ASD [6 females and 54 males, median age = 7 years, interquartile range (IQR) = 7.25] were included in our study. Before patient selection our ASD patients were screened for Fragile X syndrome and only negative cases were included in our cohort. Of our 60 patients with ASD, 58 are of European descent and 2 are Roma. Our control group for mtDNA screening consisted of 60 European adults (26 females and 34 males, median age = 28 years, IQR = 13.75) selected from our biobank [26]. All controls were healthy individuals under 45 years of age and free from addiction (alcohol, smoking, and drugs). For the interpretation of our next generation sequencing (NGS) results, we compared data from the following cohorts: patients with ASD and without mtDNA deletion, labelled non-mtdel-ASD, (6 males and 1 female, median age = 8 years, IQR = 5.5), patients with MD and mtDNA deletion, without ASD (4 males and 3 females, median age = 18 years, IQR = 19), and healthy control individuals (1 male and 5 females, median age = 27 years, IQR = 2.25). The investigated patients and controls were not related. All patients without a mtDNA deletion were considered to have non-syndromic ASD. The study design is illustrated in Table 1.

**Table 1:** The design of the study

Cohorts	M.3243 A > G, m.8993 T > C/G, m.8344 A > G	mtDNA deletion	IG NGS (51 genes)	ASD NGS (101 genes)
ASD cases (n = 60)	✔	✔	^✔a^	^✔a^
Healthy controls (n = 60)	✔	✔	^✔b^	^✔b^
mtdel-MD (n = 7)	✔	✔	✔	–

The investigated cohorts and the performed genetic analysis are shown in the Table 1. NGS testing for intergenomic panel and ASD panel has been performed in the cohort of the 10 mtdel ASD cases and in subgroup of 7 non-mtdel ASD cases and a subgroup of healthy controls (N = 6). Patients with primary mitochondrial disease (N = 7) served as further control group. All investigated person were Caucasian except 2 non-mtdel ASD cases

*ASD* autism spectrum disorder, *MD* mitochondrial disease, *mtDNA* mitochondrial DNA, *IG NGS* next generation sequencing for genes responsible for intergenomic communication, *ASD NGS* next generation sequencing for autism associated genes

^a^The 10 mtdel-ASD cases and 7 non-mtdel ASD were investigated

^b^6 cases were investigated

### Genetic analysis

DNA was isolated from peripheral blood samples from all participants using the QIAamp DNA blood kit (Qiagen, Hilden, Germany) according to manufacturer’s instructions. To identify single and multiple mtDNA deletions, long range PCR was performed as described by Remenyi et al. [27]. MtDNA single and multiple deletions were screened with long PCR in 20 μl volume: 20 pmol primers Fw 5′-TAAAAATCTTTGAAATAGGGC-3′ and Rev 5′-CGGATACAGTTCACTTTAGCT-3′, 0.2 µl Phusion DNA Polymerase (Finnzymes, Vantaa, Finland), 4 µl Phusion GC Reaction Buffer (Finnzymes, Vantaa, Finland), 0.4 µl dNTP and 12.4 μl water (qPCR grade water, AMBION). PCR program was the following: 98 °C 30 s, 30 cycles: 98 °C 10 s, 63 °C 10 s, 72 °C 3/8 min, then the last synthesis at 72 °C 7 min. Amplificates were visualised by ethidium-bromide (2% agarose) and determined with QuantityOne Software (Bio-Rad Corp. Hertfordshire, UK). The three most-frequent pathogenic mtDNA point mutations were screened by PCR–RFLP using a GeneAmp PCR System 9700 (Applied Biosystems, MA, USA) [20]. The most well-known ASD-associated genes [28] and 51 genes responsible for intergenomic communication disturbances (Additional file 1: Table S1) were investigated using NGS, which was performed on a MiSeq (Illumina, CA, USA) using the TruSight Autism Rapid Capture Kit (Illumina, CA, USA) and the SureSelect QXT Kit (Agilent Technologies, CA, USA) according to the manufacturer’s instructions. In the intergenomic panel, 16/32 samples were multiplexed in one sequencing run, whereas in the autism panel 24 samples were multiplexed in a single run using the MiSeq reagent kit v2 and 300 cycles (Illumina, CA, USA). The mean read depth was 152 × in the intergenomic gene panel and 135 × in the ASD-associated gene panel. In both panels, 20 × coverage was achieved in a minimum of 90% of target regions. Pathogenic and likely pathogenic mutations from NGS data were validated by Sanger sequencing, and segregation analysis was performed within individual families.

### Statistical and bioinformatics analysis

Chi square test with Yates correction/Fisher exact test were used to determine significant differences between patient and control groups [29]. Raw sequences were filtered with Picard tools (version 2.1.0) [30] and quality filtered reads were aligned to the hg19 reference genome with BWA-mem [31] using default parameters. Variant calling was performed using GATK HaplotypeCaller (version 3.3-0) [32] and VCF files were annotated with SnpEff (version 4.1) [33]. We analysed only those variants that were found in the canonical transcript of the gene. To identify potentially causal genetic variations, we used VariantAnalyzer, which is an in-house software developed by András Gézsi from the Budapest University of Technology and Economics [34]. This software application annotates SNPs and short indels with several types of annotations, such as their predicted function on genes using SnpEff, observed allele frequencies in several genomic projects including the 1000 Genomes Project and the ESP6500 Project, conservation scores based on PhyloP or PhastCons, predicted function of non-synonymous SNPs using dbNSFP, and disease associations with HGMD and ClinVar. By creating filter cascades based on these annotations and other information (e.g., genotypes and variant quality annotations), the software can easily be used to filter the variants through a user-friendly graphical interface. Analysis and variant calling of SureSelect libraries was performed with SureCall software (Agilent, CA, USA). First, we filtered for variants known to be disease-causing, using human gene mutation database (HGMD) Professional 2015.1 edition [35]. Second, we filtered for rare variants based on the minor allele frequency and frequency of the mutation in our NGS data repository. Since large-scale genomic data of the Hungarian population is not available, a mutation with a low minor allele frequency may also be population-specific. We labelled a variant as a rare mutation if it was present in one or two samples within our cohort and the minor allele frequency in Europeans from the 1000 Genomes and ExAC databases was less than 0.5%. It is important to note that a limitation of this method is that it may exclude identification of founder mutations and disease-associated polymorphisms. Finally, mutations were prioritized based on their predicted effects. Exonic frameshift and stop mutations were considered always damaging, whereas the effects of missense mutations were predicted using Polyphen2, SIFT (Sort Intolerant From Tolerant), or MutationTaster (MT).

## Results

**Fig. 1:**
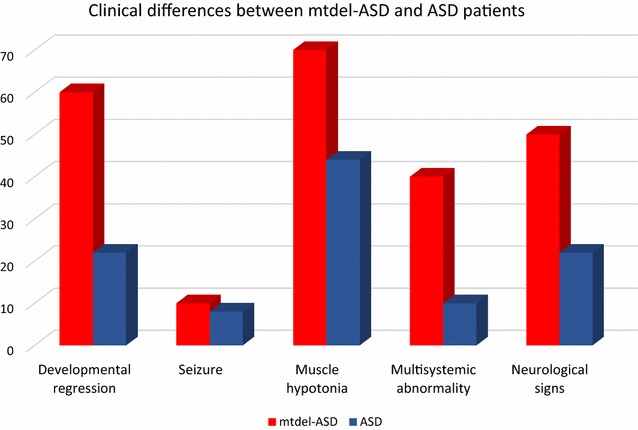
The distribution of symptoms which are common in mitochondrial disorders in the patients with mtdel-ASD and in ASD without mtdel

Sixty children with ASD (6 females and 54 males, median age = 7 years, IQR = 7.25) were investigated. In our analysed cohort of 60 patients, 29 patients were sporadic (from simplex families) and their family histories were negative for other neurodevelopmental disorders and major psychiatric or neurological disorders. The distribution of the relevant symptoms for mitochondrial disorders in mtdel-ASD and idiopathic ASD group are shown in Fig. 1. There were some differences between ASD cases with and without mtDNA deletion regarding the clinical phenotype. Developmental regression, muscle hypotonia, and additional neurological signs were most common in the mtdel-ASD cases. Multisystemic abnormality appeared also more frequently. Referring to seizures no major differences has been observed between these two groups. Family history was not available in two cases because these children were not living with their biological parents. A positive family history was found in 48% of cases, of which 20 cases (33.3%) had a family history for psychiatric disorders (bipolar disorder, depression, and schizophrenia). In four cases (6.7%), visual and hearing impairments, ataxia, complex endocrine disorders, or a combination of these factors was noted. The co-occurrence of these symptoms is an indicator for MD according to mitochondrial disease criteria (MDC) [36]. In five cases (8%), we found a positive family history for psychiatric disorders and some MDC-related symptoms were also noted.

Minor physical anomalies were identified in 44 children. All children were diagnosed with ASD based on ADI-R, and in most cases, with ADOS as well. We found that serum lactate levels and/or the lactate:pyruvate ratio supported the presence of mitochondrial dysfunction in four patients.

### Genetic investigation—mtDNA mutation screening

Mitochondrial deletions were identified in 16.6% (10/60) of our patients with ASD. Two children had multiple deletions, whereas a single major deletion was detected in the range of 2.4–7.9 kb in eight children. Detailed clinical and family history data as well as associated phenotype of the patients harbouring mtDNA deletions are shown in Table 2. An evaluation of clinical phenotype, family history, and laboratory data suggested MD in seven cases with mtDNA deletion. None of the investigated families had a previous diagnosis of primary MD. In all cases, the rate of heteroplasmy (HP) was > 20% in blood samples (Table 2). In the 60 healthy control individuals, a mtDNA deletion was found in two cases. Based on our statistical analysis, there was a significant difference in the frequency of mtDNA deletions between our ASD and control cohorts (χ^2^ with Yates correction = 4.5; p = 0.03; odds ratio = 5.8; 95% CI 1.21–27.72). Further analysis of mtDNA mutational “hotspot” regions (m.3243 A > G, m.8993 T > C/G, and m.8344 A > G) did not detect any alterations in our ASD cohort.

**Table 2:** Mitochondrial DNA deletion status and clinical data of children with ASD and mtDNA deletion

Family history	Associated diseases	Minor anomalies	Symptoms beside ASD	Laboratory results	mtDNA (HP)
MS + FS: intellectual disability, epilepsy	Chronic otitis	+	Hypoacusis, orofacial dyspraxia, intellectual disability, limb ataxia, tremor	Lactate level: 3.6 mmol/l (norm: ≤ 1.6 mmol/l), low testosterone levels, high LDH level, normal CK	Multiple (> 20%)
MS: autoimmune hypothyreosis	Gluten sensitivity	+	Attention deficit, intellectual disability, Slight macrocephaly, constipation	Lactate level: 0.6 mmol/l (norm: ≤ 1.6 mmol/l), elevated lactate/pyruvate ratio, normal CK and LDH levels	Major deletion (80%)
MS: epilepsy FS: anxiety	Tooth problems	+	Multiple congenital anomalies, coloboma, visual problems, hypotonic muscles, truncal ataxia, breathing difficulties	Lactate level: 1.9 mmol/l (norm: ≤ 1.6 mmol/l). elevated progesterone level, high LDH levels, low insulin levels	Major deletion (20%)
Mother: panic syndrome	Gastro-oesophageal reflux	+	Postnatal growth deficiency, failure to thrive, intellectual disability	Lactate level: 1.3 mmol/l (norm: ≤ 1.6 mmol/l)	Major deletion (65%)
Negative	Atopic dermatitis	−	No	Lactate levelel: 0.9 mmol/l (norm: ≤ 1.6 mmol/l)	Major deletion (35%)
Previous foetus: aborted, FS: hydrocephalus, anal atresia, MS: depression, anxiety, ptosis, OCD, carcinoma	Neonatal jaundice, strabismus	+	Microcephaly, visual problems, hypotonic muscles	Lactate level: 2.3 mmol/l (norm: ≤ 1.6 mmol/l), elevated LDH levels, normal CK level	Major deletion (20%)
Negative	No	+	Mild truncal ataxia	Lactate level: 1.2 mmol/l (norm: ≤ 1.6 mmol/l)	Major deletion (85%)
MS: bipolar disorder (3 relatives), suspected thyroid problems	Atopic dermatitis, CMV, hepatitis	+	Sensorineural hearing loss, mild myopathy, ptosis	Lactate level: 1.5 mmol/l (norm: ≤ 1.6 mmol/l), elevated LDH, norm CK level. High anti-CMV antibody titer after birth, elevated liver enzymes	Major deletion (85%)
MS + FS: PD, AD, intellectual disability; FS: suspicion of ASD	No	+	Mild truncal ataxia, calf hypertrophy	Lactate level: 1.5 mmol/l (norm: ≤ 1.6 mmol/l). Elevated lactate/pyruvate ratio, normal CK and LDH level	Major deletion (90%)
Negative	No	+	No	Lactate level: 1.2 mmol/l (norm: ≤ 1.6 mmol/l)	Multiple (> 20%)

The detected mtDNA deletion, family history, clinical data as well as associated phenotype of the ASD patients harbouring mtDNA deletions are shown in Table 2

*MS* maternal side of the family, *FS* paternal side of the family, *OCD* obsessive–compulsive disorder, *PD* Parkinson’s diseases, *AD* Alzheimer’s disease, *LDH* lactate dehydrogenase, *CK* creatine kinase, *CMV* cytomegalovirus, *mtDNA* mitochondrial DNA, *HP* ratio of heteroplasmy

### Genetic investigation—nuclear DNA mutation screening of genes responsible for intergenomic communication

Next, we focused on those patients with mtDNA deletions (mtdel-ASD) and performed nuclear DNA (nDNA) mutation screening to investigate genes involved in intergenomic communication. In this subgroup, we found one rare likely pathogenic variant and one variant with uncertain significance (VUS). The rare variants identified in two patients were both present in only one allele, however the mode of inheritance of these disorders is autosomal recessive (Table 3). In *Patient 5* (P5), the likely pathogenic heterozygous T265I mutation in the mitochondrial genome maintenance exonuclease 1 (*MGME1*) is responsible for mtDNA integrity [37]. In *Patient 9* (P9), we found a de novo VUS mutation (ClinVar ID203970) in succinate-CoA ligase alpha subunit (*SUCLG1*) in a heterozygous form.

**Table 3:** Results of the intergenomic NGS panel

Patient ID	Gene	Mutation	Zygosity	Inheritance	Clinical relevance	Polyphen2	SIFT	MT	dbSNP	ExAC	1000 Genomes/EUR AF
Patients with ASD and mtDNA deletion (N = 10)											
P5	*MGME1*	T265I	HET	AR	Likely pathogenic [37]	0.95	0.25	D	rs76599088	0.007875	0.0044/0.0139
P9	*SUCLG1*	G79D	HET	AR	Uncertain significance	0.99	0	D	n/d	n/d	n/d
Patients with ASD and without mtDNA deletion (N = 7)											
C-ASD1	*MTO1*	K321E	HET	AR	Uncertain significance	1	0.001	D	rs148667065	0.0000908	n/d
C-ASD2	*EARS2*	R99Q	HET	AR	Uncertain significance	n/d	1	D	n/d	n/d	n/d
Patients with MD and mtDNA deletion, without ASD (N = 7)											
C-MD1	*WARS2*	H151R	HET	AD/AR	Uncertain significance	0.1	0.02	D	rs150022801	0.001779	0.0008/0.003
C-MD2	*APEX1*	R202P	HET	n/d	Uncertain significance	0.6	0.01	P	n/d	0.000008242	n/d
C-MD3	*ATP5A1*	I173 V	HET	AR	Uncertain significance	0.02	0.1	D	n/d	n/d	n/d
C-MD4	*MTO1*	V517 M	HET	AR	Uncertain significance	0.03	0.1	D	n/d	n/d	n/d
C-MD5	*C10orf2*	N399S	HET	AR	Pathogenic [38]	0.896	0.09	D	n/d	n/d	n/d
	*C10orf2*	A453Q	HET	AR	Uncertain significance	0.053	0.27	D	n/d	n/d	n/d
C-MD6	*MRPL3*	S75 N	HET	AR	Pathogenic [56]	0.8	0.34	D	rs151331067	0.001606	0.0008/n/d
Healthy controls without mtDNA deletion (N = 6)											
C-H3	*EARS2*	S482 N	HET	AR	Uncertain significance	n/d	0.28	D	n/d	n/d	n/d
C-H4	*SLC25A3*	V219F	HET	AD	Uncertain significance	0.62	0.001	D	n/d	n/d	n/d

Pathogenic, likely pathogenic, and rare variants of uncertain significance detected in the 10 mtdel-ASD cases and different comparison groups are presented (benign variations are not shown)

*P* mtdel-ASD patient, *non-mtdel-ASD* ASD patient without mtDNA deletion, *MD* patient with mitochondrial disease, *H* healthy control individual, *HET* heterozygous, *AR* autosomal recessive, *AD* autosomal dominant, *n/d* no data, *SIFT* sorting intolerant from tolerant prediction database, *MT* mutation t@ster prediction database, *D* disease causing according to mutation t@ster prediction, *P* polymorphism according to mutation t@ster prediction, *ExAC* allele frequency data from exome aggregation consortium, *1000 Genomes* allele frequency data from 1000 Genomes project, *EUR AF* allele frequency in the European Super Population of the 1000 Genomes project

In our cohort of seven patients with MD (without ASD) harbouring mtDNA deletions, we found a pathogenic rare variants in two case, and rare VUS in five further cases (Table 2). In one patient the compound heterozygous state of one pathogenic and one VUS in *C10orf2* gene were detected. In cohorts without MD (patients with ASD lacking mtDNA deletion and healthy individuals) we found two–two rare VUS in genes responsible for intergenomic communications (Table 3).

Comparing the intergenomic NGS panel results for our different cohorts, we found no significant difference between mtdel-ASD cases and healthy controls with one tailed Fisher exact test (p: 0.4890 odds: 0.5, CI 0.05–4.97); and mtdel-ASD and ASD without mtDNA deletion groups (p:0.55882, Odds: 0625 CI 0.06–5.96). Likely pathogenic variant and VUS were identified in higher number in MD patients with mtDNA deletion and without ASD (p = 0.013, Odds: 0.04 CI 0.003–0.5743), in a heterozygous form (Table 3).

### Genetic investigation—nDNA mutation screening of ASD-associated genes

Using the TruSight Autism NGS panel, we detected rare SNVs in 90% (9/10) of our affected children with mtDNA deletion. Syndromic ASD was identified in a single case, *Patient 3* (P3), from our mtdel-ASD cohort. A heterozygous pathogenic mutation in chromodomain helicase DNA-binding protein 7 (*CHD7*) was found in this patient as well as a heterozygous mutation of uncertain significance in tuberin (*TSC2*). The *CHD7* rare variant regarding the ACMG guideline, fulfils the PVS1 and one PS2 criteria [38] and based on this we evaluated it as pathogenic. The patient’s phenotype and the family segregation pattern indicated this *CHD7* mutation as a de novo mutation resulting in CHARGE syndrome (OMIM 214800) [39].

In *Patient 8* (P8), we found a pathogenic nonsense mutation in 7-dehydrocholesterol reductase (*DHCR7*), which was present in only one allele.

In Patient 2 (P2) a rare mutation was detected in autism susceptibility candidate 2 (*AUTS2*), which previously was associated with syndromic ASD form. The significance of the missense mutation identified in our study is uncertain; during segregation analysis the same mutation was present in the healthy mother, however we do not know exactly the penetrance of the genetic defects of *AUTS2*. This rare *AUTS2* variant coexisted with a rare variant in retinoic acid induced gene 1 (*RAI1*) (Table 4).

**Table 4:** Results of the ASD-NGS panel

Patient ID	Gene	Mutation	Zygosity	Inheritance	Clinical relevance	Polyphen2
Patients with ASD and mtDNA deletion (N = 10)						
P1	*FOXP2*	A280T	HET	AD	Uncertain significance	0.99
P2	*RAI1*	V1565M	HET	AD	Uncertain significance	0.845
	*AUTS2*	L433P	HET	AD	Uncertain significance	1
P3	*TSC2*	K22N	HET	AD	Uncertain significance	1
	*CHD7*	Fs	HET	AD	Pathogenic [38]	n/d
P4	*RELN*	L496P	HET	AD/AR	Uncertain significance	0.98
	*KATNAL2*	R1382S	HET	AR/AD	Uncertain significance	0.99
P6	*ZNF804A*	A1108T	HET	n/d	Uncertain significance	1
P7	*RAI1*	G1070R	HET	AD	Uncertain significance	0.99
P8	*DHCR7*	W119*	HET	AR	Pathogenic	n/d
	*NHS*	R409Q	HET	XLD	Uncertain significance	1
P10	*PDE10A*	P477A	HOM	AR/AD	Uncertain significance	0.99
Patients with ASD and without mtDNA deletion (N = 7)						
C-ASD1	*SHANK2*	A1129P	HET	n/d	Uncertain significance	0.86
C-ASD2	*PON3*	S820N	HET	n/d	Uncertain significance	1
	*NRXN1*	S820N	HET	AR	Uncertain significance	0
	*CNTNAP2*	Y716C	HET	n/d	Uncertain significance	0.9
C-ASD3	*SCN2A*	L577I	HET	AD	Uncertain significance	0
C-ASD4	*NLGN4X*	Q89H	HET	XLD	Uncertain significance	0.99
C-ASD6	*GNA14*	Y287C	HET	n/d	Uncertain significance	1
Healthy controls (N = 6)						
C-H1	*ZNF804A*	A1108T	HET	n/d	Uncertain significance	1
	*NIPBL*	R765K	HET	AD	Uncertain significance	0.001
C-H2	*ZNF804A*	A1108T	HET	n/d	Uncertain significance	1
C-H3	*RELN*	A150V	HET	AD/AR	Uncertain significance	0.974

Patient ID	SIFT	MT	dbSNP	ExAC	1000 Genome/ EUR AF	
Patients with ASD and mtDNA deletion (N = 10)						
P1	0.23	D	n/d	0.000008278	n/d	
P2	0.02	P	rs368106957	0.0001819	0.0002/0	
	n/d	D	n/d	0.0002025	n/d	
P3	0.42	P	n/d	n/d	n/d	
	n/d	D	n/d	n/d	n/d	
P4	0.02	D	n/d	n/d	n/d	
	0.14	P	rs148791504	0.0009651	0.0016/n/d	
P6	0.16	P	rs112183442	0.02529	0.0158/0.0457	
P7	0.01	D	rs370633684	0.0004679	0.0004/0.00077	
P8	0.12	P	rs11555217	0.0007	n/d	
	0.31	P	n/d	0.00002282	n/d	
P10	1	P	rs61733392	0.004515	0.0024/0.006	
Patients with ASD and without mtDNA deletion (N = 7)						
C-ASD1	0.29	D	rs377255888	0.00004137	n/d	
C-ASD2	0	D	rs139856535	0.002787	0.0016/n/d	
	0.33	D	rs80293130	0.0002235	0.0002/n/d	
	0.18	D	n/d	0.00008303	n/d	
C-ASD3	0.91	D	n/d	n/d	n/d	
C-ASD4	0.1	D	n/d	n/d	n/d	
C-ASD6	1	D	rs61755085	0.001506	0.0014/0.004	
Healthy controls (N = 6)						
C-H1	0.16	P	rs112183442	0.02529	0.0158/0.0457	
	0.64	D	rs185678374	0.0005529	0.0004/n/d	
C-H2	0.16	P	rs112183442	0.02529	0.0158/0.0457	
C-H3	0.01	n/d	n/d	0.000008245	n/d	

The detected rare variants of the 10 mtdel-ASD cases, in ASD patients without a mtDNA deletion, and in healthy controls are presented (only pathogenic, likely pathogenic variations and variations with uncertain significance variations are shown)

*P* mtdel-ASD patient, *non-mtdel-ASD* ASD patient without mtDNA deletion, *MD* patient with mitochondrial disease, *H* healthy control individual, *HET* heterozygous, *AR* autosomal recessive, *AD* autosomal dominant, *n/d* no data, *SIFT* sorting intolerant from tolerant prediction database, *MT* mutation t@ster prediction database, *D* disease causing according to mutation t@ster prediction, *P* polymorphism according to mutation t@ster prediction, *ExAC* allele frequency data from exome aggregation consortium, *1000 Genomes* allele frequency data from 1000 Genomes project, *EUR AF* allele frequency in the European Super Population of the 1000 Genomes project

*The symbol of the non sense mutation in protein level

Using in silico analysis, alterations of uncertain significance were detected in ASD-associated genes in 60% (6/10) of the mtdel-ASD cases and 71% (5/7) of non-mtdel-ASD cases (Table 4). In the six control individuals only four rare VUS were detected in genes associated with ASD. The rare variant in zinc finger protein 804A (*ZNF804A*) was found in two healthy controls, indicating a variant that is likely population-specific (Table 4).

### Detailed phenotype of a patient with mtDNA deletion and CHARGE syndrome (*CHD7* mutation)

An 11-year-old male patient (P3) had multiple congenital anomalies, such as coloboma of the eyes, oxycephaly, epicanthus, convergent strabismus, mild bifid nose/broad nasal tip, low settled cup ears, mild facial asymmetry, dental dysgenesis, asymmetric chest, macroglossia, cryptorchidism, testicular hypoplasia, and atrial septal defect. He began walking at 3 years of age and suffers from obsessive hand movements, erratic behavior, and sleep disturbance. Aside from his developmental abnormalities, neurological investigation detected pes varus, severe visual impairment, bilateral ptosis, chewing difficulties, mild atrophy and weakness in the distal muscles of the extremities, and truncal ataxia. High lactate levels were detected in both serum and cerebrospinal fluid, and decreased levels of serum melatonin, calcium, and vitamin D3 were measured. A brain MRI detected hypoplastic vermis and transverse sinus on the right side. An EEG found generalized irritative signs, and VEP found an increased P100 on the left side. Brainstem auditory evoked potentials was normal. Family history identified arrhythmia, diabetes mellitus, and colon polypomatosis on the maternal side, and diabetes mellitus, arrhythmia, and dementia on the paternal side.

### Detailed phenotype of a patient with congenital cytomegalovirus infection and mtDNA deletion

The 5-year-old female patient (P8) was born at a gestational age of 39 weeks by Caesarean section with a birth weight of 3000 g. The pregnancy was complicated with a partial placental abruption at 11 weeks. Evidence of prolonged neonatal jaundice, highly elevated liver enzymes, low prothrombin level, and high IgM and IgG type anti-cytomegalovirus (CMV) antibodies led to the diagnosis of a congenital CMV infection-induced hepatic lesion. She had congenital sensorineural hearing loss, mild myopathic facies, mild ptosis, and atopic facial dermatitis. A brain MRI identified several T2 hyperintense supratentorial lesions (5–10 mm in size), which were suggested to have an infectious etiology. A heterozygous nonsense mutation in *DHCR7* and a major large single deletion in mtDNA were found. The healthy mother also harbours the detected heterozygous mutation. Cholesterol and 7-dehydrocholesterol levels of the child are in the normal range. Homozygous or compound heterozygous mutations in *DHCR7* result in Smith–Lemli–Opitz (SLOS) syndrome, which is an autosomal recessive disease. However, human CMV infection may lead to altered mitochondrial biogenesis [40]. We believe that this case demonstrates a direct interaction between genetic and environmental risk factors in some forms of ASD.

## Discussion

In this study, we provide for the first time a comprehensive genetic analysis of patients with ASD that investigates co-occurrence of the most frequent mtDNA alterations, intergenomic communication disturbances (51 genes), and 101 genes previously associated with ASD. We found co-occurrence of mtDNA deletions with ASD-associated genetic alterations, which supports the previous observation that mitochondrial alterations are frequently associated with ASD. In one patient with ASD (P3), we found a mtDNA deletion with CHARGE syndrome caused by a de novo mutation in *CHD7*. These genetic alterations in P3 were also accompanied by a *TSC2* mutation of uncertain significance. Autistic symptoms are present in approximately 30% of patients with CHARGE syndrome [39], and lactic acidosis is a rare alteration. Based on the phenotype, we conclude that the driving genetic alteration in this patient is the *CHD7* mutation, and the mitochondrial gene defect may not be the true causative factor in the etiology of the disease; however, CHD7 function is strongly ATP-dependent [41]. In addition, the associated heterozygous *TSC2* mutation is likely a modifying gene. *CHD7* is a member of the chromo-domain helicase DNA-binding (CHD) protein family and plays a role in transcription regulation through chromatin remodelling. Mutations in *TSC2* are known to cause one syndromic form of ASD; however, our patient did not develop the classic symptoms of tuberous sclerosis until recently. *TSC2* mutations may induce activation of mTORC1 leading to increased mtDNA expression and mitochondrial density. mTOR is a Ser/Thr kinase that forms complexes with numerous protein partners to regulate cell growth, mitochondrial membrane potential, and ATP synthetic capacity [42].

In *Patient 8*, we found that the mtDNA deletion was accompanied by a heterozygous pathogenic *DHCR7* mutation and a rare variant of uncertain significance in NHS actin remodelling activator (*NHS*). DHCR7 catalyses cholesterol production from 7-dehydrocholesterol, and defects in this protein cause SLOS. Furthermore, a high 7-dehydrocholesterol level results in mitochondrial dysfunction [43]. However, the significance of a heterozygous mutation in this gene is not known. We hypothesize that in the case presented here the co-occurrence of the *DHCR7* heterozygous mutation and CMV infection may play a role in changes of mitochondrial biogenesis and in the pathogenesis of autistic features. The patient was tested for SLOS; both serum cholesterol and 7-dehydrocholesterol levels were normal, which rules out the presence of typical SLOS.

Evidence of mitochondrial dysfunction in ASD was first described 19 years ago [10]. Currently, it is the most common metabolic abnormality known in ASD with a prevalence of 7.2% [14]. In a subgroup of the CHARGE (Childhood Autism Risk from Genes and Environment) study, decreased NADH activity was found in lymphocytes in 8 of 10 cases. In this cohort, only 2 of the 10 patients had mtDNA deletions and 5 patients had altered mtDNA copy numbers [12]. However, the genetic background was not clarified in 79% of the patients with ASD-MD [9]. Therefore, the possibility of secondary damage to mitochondria cannot be excluded. A small pilot study examining 12 patients with ASD described 8 mitochondrial deletions [21], which could be the result of intergenomic communication disturbances, environmental factors, or other gene–gene interactions. As is the case for many other disorders, it is still not clear whether the detected mitochondrial dysfunction in ASD is a primary or secondary event either having a key role in disease pathogenesis or is simply a downstream effect.

In our study, mtDNA deletions were identified in 16.6% of evaluated patients with ASD. During mtDNA hotspot screening and NGS analysis, no concomitant primary MD was detected. To examine whether mtDNA deletion is a primary or secondary event in ASD in our cohort, we used different comparison groups to screen the nDNA background of the mtDNA deletion. Pathogenic or likely pathogenic variants were detected in both mtdel-ASD and MD without ASD cases, all in heterozygous form. A high number of VUS in intergenomic communication genes were detected in the MD without ASD cohort (4/6), and a few rare variants were identified in patients with ASD that lacked mtDNA deletion and in healthy controls (Table 3). Homozygous or compound heterozygous mutations in *MGME1* and *SUCLG1* have been previously correlated with severe early-onset mitochondrial disorders (OMIM 615084, OMIM 245400) [44]. The importance of the presence of heterozygous mutations is not well understood. It is known in some genes responsible for intergenomic communications heterozygous mutations may result in a less severe phenotype than that found with the homozygous form [45].

The question has also been raised whether patients with MD and ASD symptoms have special characteristics. In a study by Rossignol and Frye [9], a cohort of ASD/MD children were compared to two comparison groups: children with general ASD and children with general MD. In the ASD/MD group, increased lactate and pyruvate levels, seizures, motor delays, and gastrointestinal abnormalities were significantly more prevalent compared to children with general ASD. A more balanced male:female ratio was also detected in the ASD/MD group [9]. Our results confirm the observations by Rossignol and Frye; however, in our ASD cohort with mtDNA deletion, elevated lactate levels and/or an elevated lactate:pyruvate ratio were found in only four cases, whereas most of our ASD patients with mtDNA deletion had symptoms common to MD, such as hypoacusis, muscle weakness, hypotonia, delayed motor development, and movement disorder (Fig. 1). Significant difference between mtdel-ASD and non-mtdel-ASD group was found regarding clinical phenotypes (developmental regression, muscle hypotonia, additional neurological signs and multisystemic alterations were more common in cases mt-delASD). Interestingly, the phenotypes of classic mitochondrial deletion syndromes, such as Pearson syndrome, progressive ophthalmoplegia externa, and Kearns–Sayre syndrome, were not detected in any of our patients. The family histories of mtdel-ASD children in our cohort differed from the family histories of the ASD cohort without a mtDNA deletion, since various psychiatric disorders were common among family members of mtdel-ASD cases both on maternal and paternal side. However none of the parents reached the MDC scoring cut-off value for definitive MD, which could not be independently verified because none of the family members agreed to perform muscle biopsy. MtDNA disorders are usually inherited maternally, however single mtDNA deletions are considered sporadic events with low inheritance risk, whereas multiple mtDNA deletions are the result of primary nuclear defects in genes responsible for mtDNA maintenance or nucleoside metabolism and follow Mendelian inheritance patterns [46].

Mitochondrial haplogroups were also investigated in association with ASD. Chalkia et al. found that individuals with European haplogroups designated I, J, K, X, T and U (55% of the European population) had significantly higher risks of ASD compared to the most common European haplogroup, HHV. Asian and Native American haplogroups A and M also were at increased risk of ASD [47]. In Hungary it is not rare that a person has ancient European haplotype such as T, K, and U haplotype, and rarely Asian haplotype such as B can occur as well. In some Hungarian patients the mtDNA deletion was coexisting with ancient haplotype [48].

In 90% (9/10) of children from the mtdel-ASD cohort, we found rare SNVs in ASD-associated genes (Table 4). A rare mutation was detected in *AUTS2* in which deletions are inherited in an autosomal dominant manner and are associated with neurological symptoms including intellectual disability and developmental delay [49]. In a modest study of 13 cases of ASD associated with *AUTS2* alterations, only one patient had a nonsense mutation; all the other patients had a deletion [49]. The significance of the missense mutation identified in our study is uncertain (her mother harbours the mutation as well); however, clinical symptoms of the patient correlate with the phenotype of previously published *AUTS2* mutations. This rare *AUTS2* variant coexisted with a rare variant in retinoic acid induced gene 1 (*RAI1*). The gene–gene interaction of these two alterations are hypothesized.

In addition, we found that patients had mutations of uncertain significance in forkhead box P2 (*FOXP2*), *RAI1*, phosphodiesterase 10A (*PDE10A*), katanin catalytic subunit A1 like 2 (*KATNAL2*), and reelin *(RELN*). Most of these genes play a role in cell regulation, signal transduction, and various signalling pathways, which could influence mitochondrial function. *FOXP2* is an evolutionarily conserved transcription factor that regulates the expression of a variety of genes. Mutations in this gene cause speech-language disorder 1 (OMIM 602081), which is also known as autosomal dominant speech and language disorder with orofacial dyspraxia [50]. *RAI1* acts as a transcriptional regulator of chromatin remodelling by interacting with basic transcriptional machinery [51]. *RAI1* deletion is associated with Smith–Magenis syndrome, whereas duplications are associated with Potocki–Lupski syndrome [52]. Several heterozygous mutations are also associated with Smith–Magenis syndrome [53]. In our case, we found that the typical symptoms of Smith–Magenis syndrome were not present. Mutations in *PDE10A* can affect cyclic nucleotide concentrations. This phosphodiesterase selectively catalyzes the hydrolysis of 3′ cyclic phosphate bonds in cAMP and/or cGMP. The phosphodiesterase family of proteins regulates cellular levels, localization, and duration of action of these second messengers by controlling the rate of their degradation. In addition, phosphodiesterases are involved in many signal transduction pathways and are implicated in the pathogenesis of bipolar disorder [54].

Mitochondrial dysfunction may be associated with several forms of syndromic ASD, but is also frequently related to non-syndromic cases [8, 9]. During our comprehensive analysis, we found examples of both but in most cases we did not find the causative genetic mutation that accounts for the mitochondrial dysfunction. In the examined children from the general ASD cohort (without mtDNA deletion), we found several VUS, most of which were identified in genes without previous correlation to mitochondrial dysfunction. Based on our findings, we conclude that the detected mitochondrial DNA deletions in patients with ASD in our cohort are a secondary effect. By investigating the most common mtDNA alterations and the most common nuclear genes responsible for intergenomic communications, we did not identify the clear genetic etiology in most of our cases. Therefore, further investigation and characterization is warranted.

### Limitations

We identified certain limitations in our study. We focused our investigation to analyse mutational hotspots and large mtDNA deletions and did not sequence the entire mitochondrial genome. The mtDNA mutations were analysed from blood samples; postmitotic tissue was not available. The used long PCR method detects deletions in the mtDNA with high sensitivity and low specificity. Deletions under 10% of heteroplasmy could be missed due to technical barrier, overestimation of the HP ratio is not expected. The detection of mtDNA deletion from NGS data will be in the future a new perspective, but today it is not in the everyday praxis. We used targeted NGS panels comprised of the most important genes associated with intergenomic communication and ASD. However, these panels do not include all currently associated genes and the number of these genes is continuously increasing. Finally, our healthy control group was older than our ASD cohort and had different gender ratios. However, we felt that ethically it was not appropriate to obtain biomaterial from healthy children for genetic testing. Since somatic mtDNA deletion may occur in association with ageing, and we detected the mtDNA deletion less frequently in the control group it had no impact on our data. Mitobreak Database [55] supports or presumption since deletion were present mostly only aged healthy controls, otherwise they were associated or to sporadic primary mitochondrial disorders (single deletion) or to disorders due to intergenomial gene alterations (multiple deletions).

## Conclusions

The aim of our study was to gain a better understanding of mitochondrial dysfunction in autism. We found that mtDNA alterations were more common among our cohort of patients with ASD than in control individuals. In addition, we found that the mtDNA deletion was usually not the single genetic alteration identified in ASD, but co-occurred in both syndromic and non-syndromic forms of ASD with either ASD-associated genetic risk factors and/or alterations in genes responsible for intergenomic communication. Our findings indicate a very complex pathophysiology of ASD in which mitochondrial dysfunction is not rare and can be caused by mtDNA deletion, which may be considered as de novo mutations or the consequence of the alterations of the causative culprit genes for autism or genes responsible for mtDNA maintenance.

## Additional file

**Additional file 1: Table S1.** Investigated genes responsible for mtDNA maintenance (intergenomic NGS panel).

## Abbreviations

ACMG: American College Medical Genetics
AD: autosomal dominant inheritance
AD: Alzheimer’s disease
ADI-R: autism diagnostic interview—revised
ADOS: autism diagnostic observation schedule
AR: autosomal recessive inheritance
ASD: autism spectrum disorder
ASD NGS: next generation sequencing for autism associated genes
ATP: adenosine-triphosphate
BWA: Burrows–Wheeler alignment tool
C-ASD: control ASD patient
C-H: healthy control individual
CHARGE: coloboma, heart defect, atresia choanae, retarded growth and development, genital-, ear abnormality
CHARGE: childhood autism risk from genes and environment
CHD: chromo-domain helicase DNA-binding
CK: creatine kinase
C-MD: control patient with MD
CMV: cytomegalovirus
D: disease-causing according to mutation t@ster prediction
dbNSFP: database for nonsynonymous SNPs’ functional predictions
DNA: deoxyribonucleic acid
EEG: electroencephalography
ESP6500: NHLBI GO exome sequencing project
EUR AF: Allele frequency in the European super population of the 1000 Genomes project
ExAC: exome aggregation consortium
FS: on father’s side
Fs: frameshift mutation
FS: father’s side of the family
HET: heterozygous
HGMD: human gene mutation database
HOM: homozygous
HP: heteroplasmy
IG NGS: next generation sequencing for genes responsible for intergenomic communication
IQR: interquartile range
LDH: lactate dehydrogenase
MD: mitochondrial disease
MDC: mitochondrial disease criteria
MRI: magnetic resonance imaging
MS: on mother’s side
MS: mother’s side of the family
MT: mutation taster prediction
mtdel-ASD: ASD patients with mitochondrial deletion
mtDNA: mitochondrial deoxyribonucleic acid
mTORC: mechanistic target of rapamycin complex
n/d: no data
NADH: nicotinamide adenine dinucleotide
nDNA: nuclear deoxyribonucleic acid
NGS: next generation sequencing
OCD: obsessive-compulsive disease
OXPHOS: oxidative phosphorylation
P: polymorphism according to mutation t@ster prediction
P1-10: mtdel-ASD patient 1-10
PCR: polymerase chain reaction
PD: Parkinson’s disease
PS2: pathogenic criterion is weighted as strong
PVS1: pathogenic criterion is weighted as very strong
RFLP: restriction fragment length polymorphism
SIFT: sort intolerant from tolerant amino acid substitution prediction software
SLOS: Smith–Lemli–Opitz syndrome
SNV: single nucleotide variant
VCF: variant call format
VEP: visual evoked potential
VUS: variant of uncertain significance
XLD: X-linked dominant inheritance
